# Endobronchial valve placement for intractable airleaks

**DOI:** 10.1186/1749-8090-10-S1-A271

**Published:** 2015-12-16

**Authors:** Pierre Theodore, Errol Bush, Jasleen Kukreja, Eric Seely, Antonio Gomez

**Affiliations:** 1 Department of Surgery, University of California at San Francisco, San Francisco, CA, 94143, USA; 2 Department of Medicine, University of California at San Francisco, San Francisco, CA, 94143, USA

## Background/Introduction

Prolonged alveolo-pleural and broncho-pleural airleaks can be vexing problems for Thoracic Surgeons and arise after pulmonary procedures or spontaneously: often in the setting of underlying lung disease or infection.

Patients with debilitation, HIV infection, end stage lung disease or following lung transplantation may be poor candidates for surgical intervention.

Bronchoscopic treatment with Endobronchial valves can shorten the duration of air-leaks, reducing hospital stay with an associated improvement in quality of life.

## Aims/Objectives

Our aim was conduct a trial of endobronchial valve as a therapeutic intervention for an array of clinical conditions associated with chronic alveolo-pleural fistula and airleak refractory to standard management techniques.

## Method

We performed an Institutional Review Board-approved retrospective analysis of our experience from January 2013 to April 2015.

All valves were placed after multi-disciplinary case review by pulmonologists and surgeons.

CT scans were obtained on all patients before and after valve implantation.

## Results

A total of 10 patients (7 males) with a median age of 65 (28-74) received 25 airway valves

Procedures were accomplished without evidence of valve migration.

No procedural mortality was encountered. Morbidity consisted of one post-procedure pneumonia

All 10 patients were initially treated with tube thoracostomy without success and two underwent failed attempt at surgical repair of fistula. Median time from detection of air-leak to valve placement was 14.5 days (5-45).

After endobronchial valve placement 9/10 patients had resolution of the air-leak with removal of chest tube in a medium of 3.5 days (range of 0-9 days). Median time from valve placement to discharge from the hospital was 4 days (1-12 days).

In six patients, the valves were removed without incident between 4 to 6 weeks after implantation.

## Discussion/Conclusion

Prolonged Alveolopleural fistula is a clinical challenge.

This limited experience suggests that endobronchial Valves to treat patients with prolonged air-leaks from various etiologies is effective.

Pooled data from multiple centers will permit clear patient selection criteria of patients with prolonged air-leaks.

Specifically in the group of patients unsuitable for operative intervention, endobronchial valve placement is an important management tool.

